# External quality assessment schemes in bacteriology support public health in Germany—results from 2006 to 2023

**DOI:** 10.3389/fmolb.2024.1395410

**Published:** 2024-05-17

**Authors:** Marc Lindenberg, Sabine Waldmann, Sebastian Suerbaum, Dirk Schlüter, Stefan Ziesing

**Affiliations:** ^1^ Institute for Medical Microbiology and Hospital Epidemiology, Hannover Medical School, Hannover, Germany; ^2^ German Center for Infection Research (DZIF), Munich, Germany; ^3^ Max von Pettenkofer Institute, Faculty of Medicine, Ludwig-Maximilians-Universität, Munich, Germany; ^4^ National Reference Center for Helicobacter Pylori, Munich, Germany; ^5^ German Center for Infection Research (DZIF), Partner Site Hannover-Brunswick, Hannover, Germany; ^6^Management of External Quality Assessment Schemes Bacteriology, Instand e.V., Düsseldorf, Germany

**Keywords:** microbiology, bacteriology, external quality assessment, public health, antimicrobial susceptibility testing (AST), identification methods bacteriology

## Abstract

External Quality Assessment schemes (EQAS) are mandatory to ensure quality standards in diagnostic methods and achieve laboratory accreditation. As host institution for two German culture-based bacteriology EQAS (RV-A and RV-B), we investigated the obtained data of 590 up to 720 surveys per year in RV-A and 2,151 up to 2,929 in RV-B from 2006 to 2023. As educational instruments, they function to review applied methodology and are valuable to check for systemic- or method-dependent failures in microbiology diagnostics or guidelines. Especially, containment of multi-resistant bacteria in times of rising antibiotic resistance is one major point to assure public health. The correct identification and reporting of these strains is therefore of high importance to achieve this goal. Moreover, correct antimicrobial susceptibility testing (AST) *per se* is important for selecting appropriate therapy, to restrict broad-spectrum antibiotics and minimize resistance development. The reports of participating laboratories displayed a high level of correct identification results in both schemes with mostly consistent failure rates around 2.2% (RV-A) and 3.9% (RV-B) on average. In contrast, results in AST revealed increasing failure rates upon modification of AST requirements concerning adherence to standards and subsequent bacterial species-specific evaluation. Stratification on these periods revealed in RV-A a moderate increase from 1.3% to 4.5%, while in RV-B failure rates reached 14% coming from 4.3% on average. Although not mandatory, subsequent AST evaluation and consistent reporting are areas of improvement to benefit public health.

## 1 Introduction

Conventional culture-based identification of bacteria and subsequent antimicrobial susceptibility testing (AST) remain the gold standard and represent the largest part of bacteriological diagnostics in medical microbiology, although molecular biological methods have and will further improve the identification of bacterial pathogens. However, at present, AST as a central task of every diagnostic microbiological-bacteriological laboratory can only be performed adequately by culture-based techniques but not by molecular biological methods including whole genome sequencing (Jorgensen and Ferraro, 2009; Turnidge et al., 2023). Due to the outstanding importance for the detection of infections and selection of suitable therapeutic options based on AST - especially in times of constantly increasing antibiotic resistance–the applied methods are subject to not only internal laboratory quality assessment but also external quality assessment, which is mandatory in Germany. The public health system is also dependent on assured and constantly evolving quality in bacteriology especially concerning i) reliable and fast identification for reporting of notifiable pathogens, ii) rapid and reproducible AST in accordance with standards to inform clinicians about safe and efficient treatment options and to prevent unnecessary usage of broad-spectrum substances, iii) and an up-to-date and uniform nomenclature, as well as antibiotic-susceptibility assessment standards such as EUCAST and CLSI to assure correct communication between key players of the healthcare system.

Different national laws and guidelines oblige microbiology laboratories to participate in External Quality Assessment schemes (EQAS). In Germany, the Federal Medical Council issues these binding guidelines for all medical laboratories (RiliBÄK) (Bundesaerztekammer, 2023). Reference institutions including Instand e.V. manage these EQAS in collaboration with host diagnostic microbiological laboratories. For laboratories performing bacteriology diagnostics, successful participation in EQAS, at least once a year, is a prerequisite to receive reimbursement of costs for diagnostic procedures with the respective cost bearers. In Germany, INSTAND e.V. has been performing EQAS in bacteriology with fast-growing organisms since 2006 with the Institute for Medical Microbiology and Hospital Epidemiology of Hannover Medical School, Hannover, Germany as host institution. The host institution acts as scientific management partner with selection of suitable bacterial strains, production of specimens, evaluation, and commenting of results for each survey. Instand e.V. organizes the surveys with respect to the shipment of specimens, both nationally and internationally, recording the results and providing them to the host laboratory for final evaluation. Successful participation in EQAS is a prerequisite to obtaining accreditation, as stated in the International Standard ISO 15189:2022 (ISO, 2022).

**FIGURE 1:**
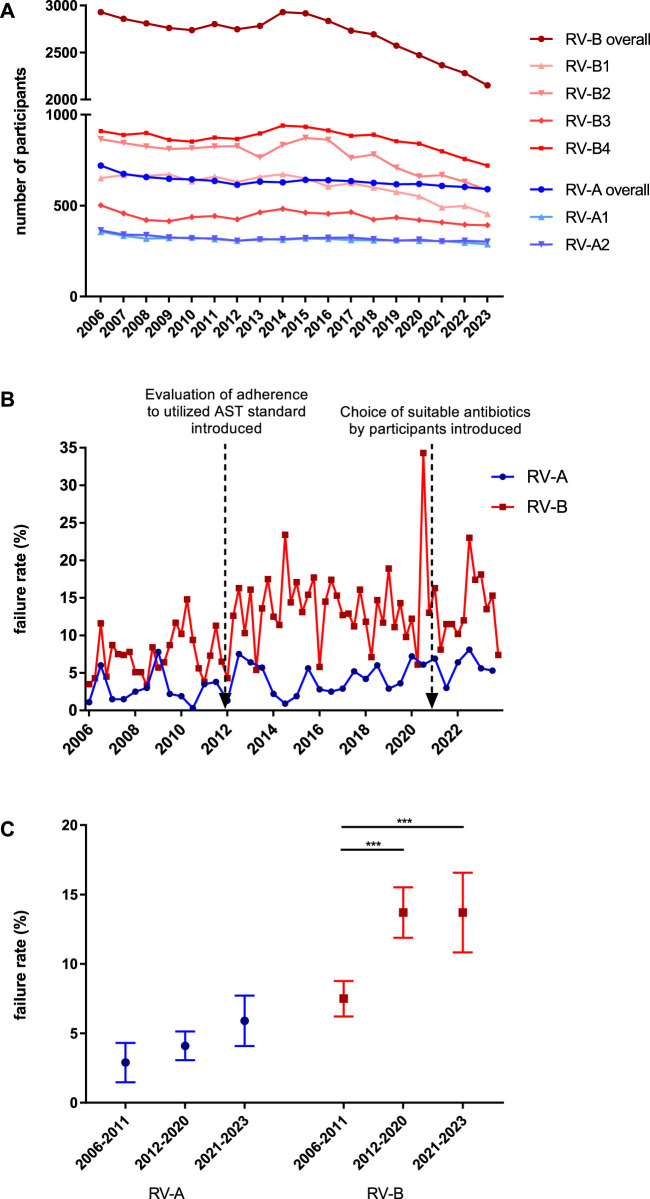
Analysis of participants and passing rates in bacteriology EQAS RV-A and RV-B. **(A)** Number of participants in both EQA schemes from 2006 to 2023 for the respective annual dates. **(B)** Failure rates in RV-A (blue) and RV-B (red) EQAS at the respective dates with red arrows indicating time points of the described modifications in the EQAS. **(C)** Overall failure rates in RV-A and RV-B categorized for the periods between the aforementioned modifications in the EQAS. Depicted are mean ± SD for 12 (2006–2011), 18 (2012–2020), and 6 (2021–2023) data points in RV-A and 24 (2006–2011), 36 (2012–2020), and 12 (2021–2023) in RV-B.

In Germany, diagnostic bacteriology is performed by specialized laboratories but also by outpatient practitioners who provide diagnostics for their specialty, and here urologists are by far the largest group by numbers. The German guidelines consider the diagnostic differences leading to two different EQA schemes. Bacteriology “Ringversuch A” (RV-A), directed to specialized microbiology laboratories and sent out twice a year with five bacterial samples, and “Ringversuch B” (RV-B), directed to outpatient practitioners and sent out four times a year with three probes containing urogenital pathogens or commensals but not restricted to bacteria. In both schemes, slow-growing bacteria like mycobacteria, which are subject to separate EQAS, are excluded. Besides direct quality assurance, other beneficial aspects of EQA are that the host institution issues a certificate upon successful participation, which is mandatory to hand costs to the respective cost-bearers. In addition, the EQA host institution is obliged to report abnormalities to the respective authorities for instance to the Federal Institute for Drugs and Medical Devices. Of note, every EQAS round is also a test for the issued diagnostic guidelines for instance the breakpoint tables. Finally, EQAS are educational and can spread new knowledge on nomenclature, current epidemiology, clinical relevance of microorganisms, newly described resistance mechanisms, and phenotypic appearances that may lead to misinterpretation. The appended commentaries in the result reports are highly valuable in spreading knowledge. Within this study, we analyzed a highly consistent 288 to 364 laboratory reports per survey in RV-A and a more varying 392 to 940 per survey in RV-B as a large and representative database (Figure 1A).

## 2 Materials and Methods

### 2.1 Identification part in EQAS bacteriology

In RV-A directed to specialized microbiology laboratories, the host laboratory sends out five specimens of bacterial strains twice a year with one specimen per year as a mixture of two strains. Participants need to identify strains on genus and species level and obtain one point for correct identification per level. To pass this category at least 80% of all points need to be gained per survey. The 80% cut-off value has already been defined since at least 1992 and has not been changed with the takeover of the EQAS by the current host laboratory.

In RV-B, performed four times a year and directed to laboratories performing bacteriology within the scope of their respective profession, which is overwhelmingly urology, the focus is on urogenital pathogens and commensals. Here, three specimens are sent but are not strictly limited to bacteria but can also contain yeast strains, without consequent susceptibility testing. Four of the six points must be gained in this category to pass in RV-B per survey.

Reference results are obtained from a consortium of 16 highly qualified microbiology laboratories, which are referred to as target value laboratories (TVL) from here on. Not all TVL take part in every survey. TVL are suggested by the host laboratory and have to be accepted by the Federal Medical Council. Most of them have acted as TVL for more than 2 decades.

### 2.2 Antimicrobial susceptibility testing (AST) part in EQAS bacteriology

From a table of 16 antibiotics for RV-A and 15 antibiotics for RV-B, the participants have to choose and report those suitable to treat the identified bacterial species in accordance with the used AST standard. For each specimen a minimum count of antibiotics (approximately three-quarters of the maximum number assessable) to be tested is defined by the host institution of the EQAS, dependent on the number of antibiotics evaluable with respect to the utilized AST standard. Participants need to test the identified bacteria and report interpreted results as susceptible S), intermediate respectively susceptible at increased dosing (I, the latter definition valid for EUCAST since 2019), or resistant R). For every substance, a full point is gained by meeting one interpretation of the set point range, while half a point is granted in case of “minor errors”; I instead of R, for example, (Turnidge et al., 2023). In RV-A and RV-B, both the given minimum number of antibiotics and 85% of all points for correct results are needed to pass. AST is performed in parallel to the participants by the TVL three times to account for technical variability, which can be method-dependent. TVL report only one final result to the host laboratory and are asked to deliver results for at least two combinations of technique–disk diffusion or MIC determination–and AST standard. Based on these values the set point range is determined. Depending on the scattering of TVL results the target value is set usually to one level as S, I, or R. Following a defined algorithm, in case of broader scattering more than one level might be accepted.

### 2.3 Timeline of modifications

Initially in 2006, when the Institute of Medical Microbiology and Hospital Epidemiology of Hannover Medical School took over the management of the EQAS, participants were required to identify the strain on genus and species level and test susceptibility against at least six out of eight defined antibiotics by disk diffusion according to the German Institute for Standardization (Deutsches Institut für Normung, DIN) standard. At first, we re-defined the required AST panel with respect to the strain identified as being Gram-positive or Gram-negative. From 2012 onwards, participants were required to report the utilized AST standard, while the ones of DIN, Clinical and Laboratory Standards Institute (CLSI), and European Committee on Antimicrobial Susceptibility Testing (EUCAST, 2024) were accepted. While DIN was excluded in 2014 as being outdated and discontinued, EUCAST modified by recommendations of the national antibiotic susceptibility testing committee Germany (NAK) for certain substances was accepted from 2016 onwards (EUCAST and EUCAST + NAK were summarized for data analysis in this paper). Reported results in AST were evaluated in correlation to the reported and utilized standard.

### 2.4 Data collection

Participants are asked to report their results on standardized questionnaires in each EQAS round.

While up to 2019 paper-based reports had to be handed in by the participants, from then onwards an online form is mandatory. This online form made it feasible to obtain additional data on pathogenicity, extended bacterial typing, reporting obligations, detected mechanism of resistance, and, as a German specialty, multi-resistant phenotypes in Gram-negative rods which are used for management in hospital hygiene and, in part, have to be reported to the public health authorities.

### 2.5 Data analysis

We analyzed the reported results of participating laboratories from 2006 until 2023. As the EQA definitions and requirements changed over time, different analysis topics span different time frames defined by changes in the requirements for susceptibility testing. From 2006 to 2011: Disk diffusion according to DIN only. From 2012 to 2021: Reporting the utilized guideline for result interpretation (DIN, CLSI, EUCAST, EUCAST plus NAK), both disk diffusion and MIC techniques possible. From 2021 onwards: Participants have to select the antibiotic substances to be reported in accordance with the utilized AST standard.

### 2.6 Statistics

GraphPad Prism Version seven was used to determine significance of results. Figure legends describe statistical tests run on respective data sets. One-way-Analysis of variance (ANOVA)-test was used if not indicated differently and means are given as ± *s*.d. with *p* values considered significant as follows: * = *p* < 0.05; ** = *p* < 0.005 and *** = *p* < 0.0005.

## 3 Results

### 3.1 Analysis of participants and passing rates in bacteriology EQA schemes RV-A and RV-B

For public health, a high standard of microbiological diagnostics and a solid data basis is required and EQAS evaluate this for the participating laboratories. Since 2006, we recognized an almost constant number of participants in RV-A, directed at specialized microbiological laboratories at both time points per year. In contrast, the number of participants in RV-B varied greatly on the four annual dates. The last date of each year had by far the highest number of participants. From 2006 to 2023, the overall failure rates in these EQAS were in the range of 0.3%–8.1% in RV-A (mean 4.0% ± 2.23%) and 3.3%–34.3% in RV-B (mean 11.6% ± 5.39%). Failure rates increased for periods following modifications to the EQAS with respect to AST evaluation (details in Material and Methods) (Figure 1B). Further analyzing the results of the different periods with increasing demands on participants, we found them significantly increased for RV-B and trending in the same direction on a lower overall level for RV-A (Figure 1C). Additionally, failure rates were substantially higher in RV-B as compared to RV-A illustrating better diagnostic quality of RV-A participants according to the EQAS criteria.

### 3.2 Identification of bacteria, development of identification success, and methods used

**FIGURE 2:**
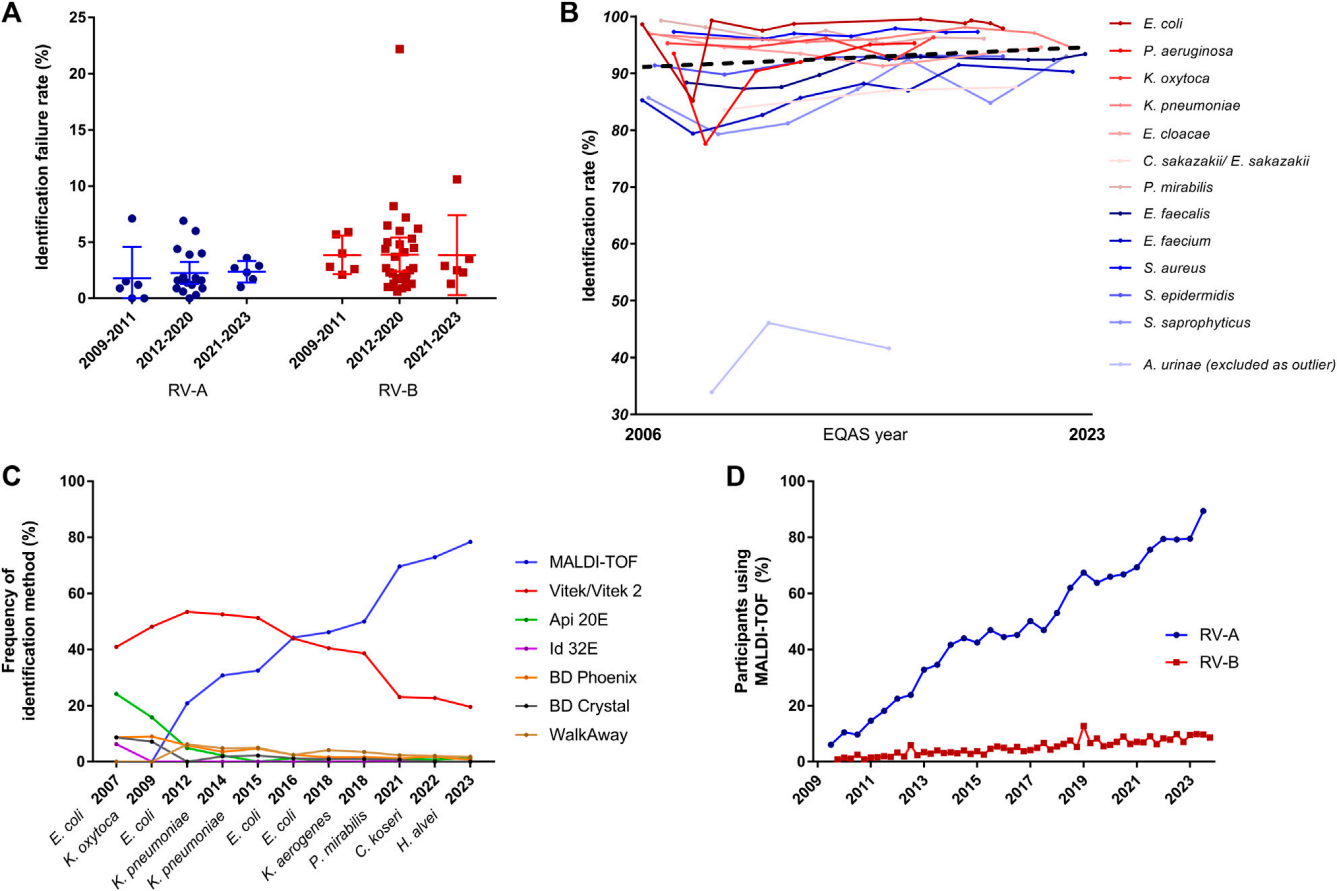
Analysis of identification rates over time. **(A)** Failure rates in identification in RV-A and RV-B categorized in periods with regard to modifications in the EQAS. Dots represent respective overall failure rates per EQAS survey. **(B)** Identification rate for bacterial species being sent out at least three times from 2009 until 2023 in RV-B. Gram-negatives depicted in shades of red, gram-positives in shades of blue according to the legend in the graph, while the dotted line represents the regression line over all data points excluding rates for *A. urinae*. Regression analysis gave a slope of 0.049 (95% CI: 0.009–0.108). **(C)** Analysis of utilized identification methods for Enterobacterales in RV-A for the indicated time points. **(D)** Frequency of MALDI-TOF as identification method among RV-A (blue) and RV-B (red) participants.

To determine the reasons for increased failure rates upon EQAS modification, we further analyzed failure rates in identification and AST separately as both had to be passed by participants. Correct identification of bacteria is a prerequisite for correct AST considering species-specific breakpoints. The analysis of identification results from 2009 to 2023 showed no significant changes over time for both RV-A and RV-B and also between RV-A and RV-B with failure rates as low as 2.1% (±1.6%) in RV-A and 3.9% (±3.30%) in RV-B in this EQAS category (Figure 2A). However, as successful identification rates varied between different bacterial species, we checked for improvements on a bacterial species-dependent level. In RV-A considerable improvements in the accuracy of identification of rarely detected and challenging bacterial species were already shown by the EQAS of the time since their introduction in 1982 (Schaal, 1994). Since 2006, we found significant improvements only for a comparatively small number of species (Table 1). For some species, occasionally a slight decline in identification rates was found compared to previous EQAS rounds. In most of these cases, affected strains were part of a germ mixture consisting of two bacteria, where the potential problem of retrieval may add to the increased failure rate.

**TABLE 1:** Identification rate for selected species in RV- A. Displayed species are selected due to their relevance with respect to guideline adherence, taxonomic changes, frequency of isolation, and culture conditions. (*: strain has been part of a germ mixture).

Species	Date	Rate [%]	Date	Rate [%]	Date	Rate [%]
*Arcobacter butzleri*	1–2009	60.6	1–2019	73.5		
*Bacillus pumilus*	1–2011	77.5	1–2022	84.8		
*Bacteroides fragilis*	1–2006	95.2	1–2021	94.4	2–2023	94
*Campylobacter jejuni*	1–2007	91.6	2–2020	92.7		
*Clostridioides (Clostridium) difficile*	2–2006	99.2	2–2015	94.7	1–2019	94.8
*Clostridium tertium*	2–2006	88.4	1–2022	91.6		
*Corynebacterium belfantii, C. diphteriae complex, C. rouxii; toxin-negative*	2–2014	97.8	1–2023	96.5		
*Cronobacter (Enterobacter) sakazakii*	2–2007	100	1–2021	99.7		
*Cutibacterium (Propionibacterium) acnes*	2–2008	95.3	1–2009	87.1*	2–2018	92.7
*Eikenella corrodens*	2–2007	97.3	1–2023	97.9		
*Finegoldia magna*	1–2011	95.6	1–2021	92.5*		
*Granulicatella (Abiotrophia) adiacens*	2–2012	89.2	1–2020	91.5		
*Haemophilus influenzae*	2–2011	98.4	1–2016	98.1		
*Kingella kingae*	2–2008	93.2	2–2022	93.5		
*Klebsiella (Enterobacter) aerogenes (Klebsiella mobilis)*	2–2010	98.4	2–2018	99.7		
*Listeria monocytogenes*	2–2010	99.4	2–2016	98.5	2–2020	98.1
*Mammaliicoccus (Staphylococcus) sciuri*	1–2019	98.4	1–2023	100		
*Micrococcus luteus*	2–2007	99.7	1–2015	90.3*		
*Pasteurella multocida*	1–2012	94.1	1–2013	93.3		
*Rahnella aquatilis*	1–2008	98.1	1–2017	96.8		
*Serratia marcescens*	1–2007	99.4	2–2009	96.3*	1–2017	99.4
*Staphylococcus (Peptococcus) saccharolyticus*	2–2010	94.7	1–2014	67*		
*Staphylococcus caprae*	1–2017	96.8	1–2022	98.3		
*Staphylococcus lugdunensis*	1–2008	98.1	1–2016	98.4		
*Staphylococcus schleiferi*	2–2011	100	2–2018	98.4		
*Streptococcus canis*	2–2017	81.5	2–2021	89.4		
*Streptococcus dysgalactiae*	1–2016	93.1	2–2019	93.2		
*- dysgalactiae equisimilis*		68.8		64.2		
*Streptococcus gallolyticus* sp. *Gallolyticus*	2–2009	98.2	1–2014	99.4		
*Vibrio vulnificus*	2–2008	98.5	1–2019	97.1		
*Weeksella virosa (CDC group IIf, Flavobact. sp?)*	2–2012	88.9	1–2020	81.4		
*Yersinia enterocolitica*	2–2008	99.7	1–2014	99.7	1–2022	98.6

Moreover, we analyzed the identification success for bacterial species being sent out repeatedly (in both RV-A and RV-B. While participants in RV-A were overall more successful than in RV-B, for common urogenital pathogens an overall high standard of identification rates was observed (Table 2). However, bacterial species rarely causing urinary tract infections but need to be identified in accordance with diagnostic guidelines were more challenging for participants in RV-B (Table 3).

**TABLE 2:** Comparison of identification rates in RV-A and RV-B for frequent urogenital species.

Species	RV-A			RV-B		
	Surveys [n]	Mean [%]	Range [%]	Surveys [n]	Mean [%]	Range [%]
Gram-negative						
*Escherichia coli*	6	99.7	99.3–100	10	97.4	85.2–99.3
*Pseudomonas aeruginosa*	4	98.5	96.4–99.7	10	92.3	77.6–96.9
*Klebsiella pneumoniae*	2	99.1	98.4–99.7	8	96.3	94.5–98.1
*Proteus mirabilis*	4	99.8	99.1–100	7	96.9	95.2–99.3
*Enterobacter cloacae*	2	97.6	96.1–99.1	5	94.2	91.3–97.1
Gram-positive						
*Enterococcus faecalis*	3	98.9	98.4–99.4	10	91.0	87.3–93.4
*Enterococcus faecium*	1	98.7	-	8	86.3	79.4–91.5
*Staphylococcus aureus*	6	99.6	99.2–100	7	97.0	96.0–97.9
*Staphylococcus saprophyticus*	1	98.7	-	7	86.2	79.3–93.0
*Staphylococcus epidermidis*	1	98.8	-	5	92.0	89.8–93.1

**TABLE 3:** Identification rates for rarely found species in RV-B.

Species	Date	Rate [%]	Date	Rate [%]	Date	Rate [%]	Date	Rate [%]	Date	Rate [%]
*K. oxytoca*	1–2007	95.3	2–2010	94.6	2–2013	96.2	1–2016	92.6	3–2017	96.3
*Klebsiella aerogenes*	3–2016	-	2–2019	37.9						
*“Enterobacter” aerogenes*		92.5		57.5						
*Cronobacter sakazakii*	2–2009	1.2	4–2015	56.7	4–2020	76.1				
“*Enterobacter” sakazakii*		82.4		30.3		15.0				
*Pantoea agglomerans*	3–2009	82.9	3–2012	86.6	4–2022	91.1				
*Serratia marcescens*	2–2008	95.0	4–2012	94.0	3–2021	91.7				
*Corynebakterium urealyticum*	2–2008	44.7	3–2012	31.6	3–2017	37.9	2–2023	58.6		
*Staphylococcus lugdunensis*	2–2016	48.8	1–2022	52.2						
*Micrococcus luteus*	2–2010	83.6								
*Aerococcus urinae*	4–2008	33.9	1–2011	46.1	4–2015	41.6				
*Actinobaculum (Actinotignum) schaalii*	3–2020	16.2								
*Lactobacillus*	1–2007	41.5	1–2012	55.4						
*rhamnosus*		5.5		8.3						

As the failure rates in identifications among participants in RV-A were very low, we focused on RV-B results to track changes over time. To account for the educational aspect of the EQAS, we analyzed the identification rate of bacterial species in RV-B being sent out three or more times between 2006 and 2023. We observed a slight trend to increased accuracy in identification rates, however, the slope of the fitted regression line was not significantly different from zero (*p* = 0.09) (Figure 2B). 

Nonetheless, we asked for methodological improvements over time. Therefore, we analyzed developments in the participant’s identification methods used for Enterobacterales identification during different EQAS rounds. While RV-A laboratories overwhelmingly changed to rely on Matrix-associated-laser-desorption-ionization and time of flight (MALDI-TOF) analysis, a technique considered to be fast and of high accuracy (Dingle and Butler-Wu, 2013), this technique is still not widely used by RV-B participants (Figures 2C, D).

### 3.3 Adherence to nomenclature

**FIGURE 3:**
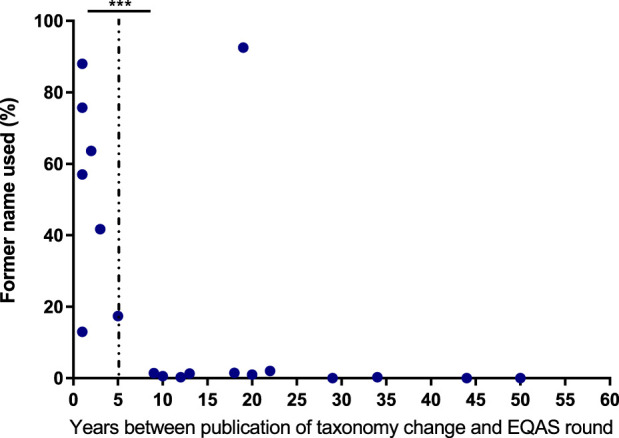
Adherence to new taxonomy A Data display the frequency of bacterial species undergoing taxonomy changes being reported with the former name correlated to the time between publication of the new name and the respective EQAS round. The dotted line indicates categorization in more than 5 years since publication and equal or less for the respective data points. Significance was tested by a chi-square test. *** = *p* < 0.005.

In view of consistent reporting of diagnostic microbiological results to clinicians and health authorities, correct identification and also the use of current terminology is a desirable goal. Therefore, we studied results from EQAS strains with changes in taxonomy (Table 4). For participants, there are usually no drawbacks when adhering to outdated names, as both new and old names are accepted in the EQAS, and hence this topic is not recapitulated in the failure rate analysis. Categorizing the time since the publication of the new name and the respective EQAS round, we found a significant correlation between the updated taxonomy being reported and a period greater than 5 years since the renaming (Figure 3).

**TABLE 4:** Reported names for bacteria with changes in taxonomy in RV-A.

RV-A	Taxonomy		
	Former name	New name	year of renaming
2–2007: *Aggregatibacter (Haemophilus) aphrophilus*	75.7	17.3	2006
2–2007: *Pantoea (Enterobacter) agglomerans*	1.5	94.4	1989
1–2009: *Cupriavidus (Ralstonia, Wautersia) pauculus*	17.4	73.3	2004
2–2010: *Staphylococcus (Peptococcus) saccharolyticus*	0	94.7	1981
2–2010: *Enterobacter aerogenes (Klebsiella mobilis)*	0	98.4	1960
2–2011: *Raoultella (Klebsiella) planticola*	0.6	81.3	2001
2–2012: *Granulicatella (Abiotrophia) adiacens*	0.3	88.9	2000
2–2012: *Moraxella (Branhamella) catarrhalis*	0	99.0	1968
2–2016: *Actinobaculum (Actinotignum) schaalii*	13.0	73.1	2015
2–2017: *Paeniclostridium (Clostridium) sordellii*	88.0	1.2	2016
2–2018: *Cutibacterium (Propionibacterium) acnes*	63.6	29.1	2016
2–2018: *Klebsiella (Enterobacter) aerogenes*	57.0	42.7	2017
1–2020: *Weeksella virosa* (CDC group IIf, *Flavobacterium*)	0.3	81.7	1986
1–2020: *Granulicatella (Abiotrophia) adiacens*	0.3	91.8	2000
1–2021: *Cronobacter (Enterobacter) sakazakii*	1.3	98.7	2008
1–2021: *Finegoldia magna (Peptostreptococcus)*	2.0	92.5	1999
1–2021: *Pantoea (Enterobacter) agglomerans*	1.6	98.0	1989
2–2021: *Raoultella (Klebsiella) ornithinolytica*	1.0	97.7	2001
2–2022: *Delftia (acidovorans) tsuruhatensis*	92.5	2.3	2003
1–2023: *Empedobacter (Wautersiella) falsenii*	1.4	59.4	2014
2–2023: *Lacticaseibacillus (Lactobacillus) rhamnosus*	41.7	55.3	2020

### 3.4 Results of antimicrobial susceptibility testing (AST)

**FIGURE 4:**
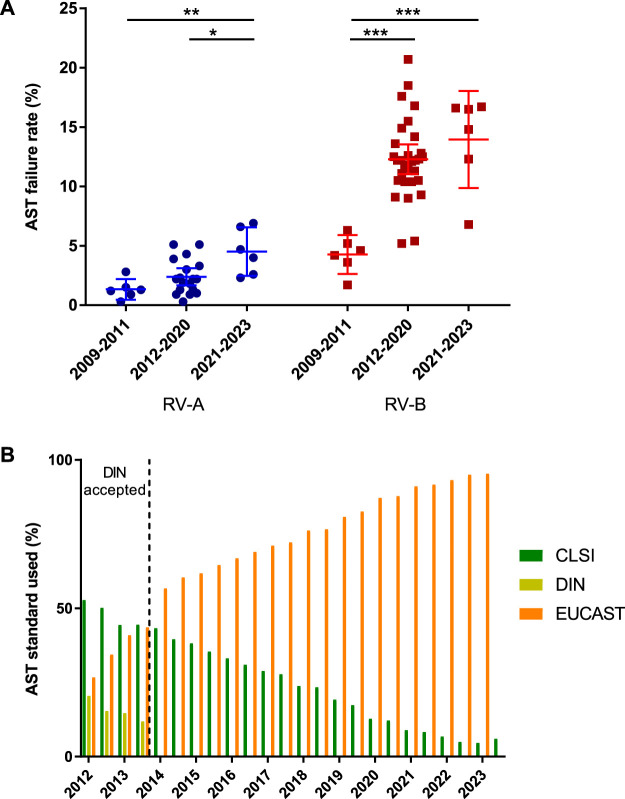
Analysis of antimicrobial susceptibility testing (AST). **(A)** Failure rates in AST for RV-A and RV-B categorized in periods with regard to modifications in the EQAS. Depicted are mean ± SD for 6 (2009–2011), 18 (2012–2020), and 6 (2021–2023) data points in RV-A and 6 (2009–2011), 36 (2012–2020), and 6 (2021–2023) in RV-B. **(B)** Frequency of AST standard used by participants in RV-A from 2012 to 2023. Color schemes of the different standards according to the figure legend.

As the overall increase in failure rates was not attributable to the identification rates, we analyzed the AST results of participants. The specialist microbiological laboratories in RV-A documented a high level of quality in AST, while RV-B participants showed poorer accuracy rates, which also scattered over a wider range. The two considerable changes to the requirements in this part of the EQAS led to increased failure rates in both series, with considerably stronger effects in RV-B (Figure 4A).

With respect to the requirements of the public health system, the performance of the participants in the identification of resistance mechanisms is of particular interest; especially as the initiation of hygiene measures to prevent pathogen spread depends on these results. While not obligatory and not evaluated in the EQAS - as no international standard is applicable - participants had the opportunity to indicate identified bacteria and phenotypic AST combinations with the respective acronym. The reliability of detecting and reporting oxacillin resistance in *Staphylococcus aureus* (MRSA), vancomycin resistance in enterococci (VRE), or Extended-Spectrum-Betalactamase (ESBL) expression in Enterobacterales was high in RV-A (data not shown). However, in RV-B, only a minority of strains were reported with the respective acronyms, even though phenotypically characterized as resistant. Some showed an increase in reporting over time anyway (Table 5).

**TABLE 5:** Voluntary reporting on resistance mechanisms for repeatedly sent bacteria in RV-B.

Species: Resistance mechanism	EQAS round	Rate (%)	EQAS round	Rate (%)	EQAS round	Rate (%)	EQAS round	Rate (%)	EQAS round	Rate (%)	EQAS round	Rate (%)
*E. coli*: ESBL	1–2008	11.7	4–2010	19.3	1–2012	26.3	1–2017	19.7	2–2020	28.9		
*K. pneumoniae*: ESBL	2–2006	8.4	3–2008	18.3	1–2011	16.5	3–2012	31.8	2–2015	24.8		
*P. mirabilis*: ESBL			3–2008	16.2	2–2013	18.8						
*E. faecium*: VRE	1–2008	8.3	4–2010	14.3	2–2012	14.4	4–2014	13.2	3–2016	15.4	1–2023	19.2
*S.aureus*: MRSA	2–2007	20.2	4–2010	23.7	1–2012	37.8	2–2014	30.0	1–2016	36.1		
*S.aureus*: MRSA - specified as mecC									1–2018	30.7 1.8	2–2019	5.2 0.8

In Germany, carbapenem resistance due to carbapenemases, especially in Enterobacterales, has only been an epidemiological problem since 2010 (Albiger et al., 2015). We had a look at the performance of the RV participants over time concerning carbapenemase detection. Table 6 summarizes the results on various carbapenem-resistant bacteria in RV-A and RV-B with green color indicating increases in reporting for repeatedly sent-out strains.

**TABLE 6:** Comparison of results in carbapenemase characterization between RV-A and RV-B.

Species: Resistance mechanism	Target value	RV-A			RV-B		
			Reported as…			Reported as…	
		EQAS round	Any carbapenemase (%)	Target value (%)	EQAS round	Any carbapenemase (%)	Target value (%)
*Acinetobacter pittii:* GIM-1	MBL	A2-2017	23.8	5.5	N/A		
*Citrobacter freundii:* VIM-1	MBL	A1-2019	39.0	26.4	B3-2019	11.6	7.1
*Escherichia coli:* OXA-181	OXA-Type	A2-2016	43.3	17.9	B2-2023	12.5	9.9
*Escherichia coli:* VIM-1	MBL	A2-2012	54.1	40.7	B1-2013	4.6	2.3
		A2-2020	74.8	59.4	B1-2019	5.9	3.0
					B1-2022	10.2	6.6
*Klebsiella pneumoniae:* KPC	KPC	N/A			B3-2013	7.3	3.0
					B1-2023	12.8	9.5
*Klebsiella pneumonia:* NDM	MBL	A1-2015	25.9	16.9	N/A		
*Klebsiella pneumonia:* OXA-48	OXA-Type	A1-2014	75.3	21.8	B1-2020	13.6	6.4
*Klebsiella (Enterobacter) aerogenes:* AmpC + porine loss	AmpC + porine loss	A2-2018	8.2	19.3	B2-2019	3.4	2.1
*Proteus mirabilis:* NDM	MBL	A1-2016	19.6	16.4	B2-2021	10.6	6.7
*Pseudomonas aeruginosa:* VIM-2	MBL	A2-2016	25.7	17.9	B2-2017	5.0	2.2
					B2-2022	9.0	6.5

Since the detection of carbapenemases can be challenging, we analyzed the results of a New Delhi carbapenemase (NDM)-producing *Proteus mirabilis* sent out in RV-A1 in 2016. Table 7 compares the documented MIC values reported by target value laboratories (TVL) and categorizes results from participants stratified according to CLSI and EUCAST (Table 7). The MIC values varied widely and both the TVL and the participants rated a similarly high proportion of the tests as “S" or “I”, while NDM-carrying bacteria are usually considered phenotypically carbapenem-resistant. We found that more than 30% of EUCAST participants reported the strain meropenem susceptible or intermediate, while this was true for about 10% of CLSI users only. This example illustrates the difficulty in detecting carbapenemase expression solely upon AST and puts the results of only 16.4% of the participants characterizing an NDM- or metallo-ß-lactamase, 19.6% reporting a carbapenemase without further characterization and 64.1% of participants indicating no resistance mechanism at all, in perspective. Moreover, evaluation is highly dependent on the utilized AST standard in this case.

**TABLE 7:** NDM-expressing *Proteus mirabilis* RV-A1-2016: Variance in meropenem MICs and classification determined by TVL and EQAS participants.

Meropenem							Rate (%)		
MIC (mg/L)	0.5	1	2	4	8	≥16	S	I	R
CLSI-TVL n)	**1**		**1**	**3**	**2**	**9**	6.3	6.3	87.5
- participants n)	**3**		**5**	**70**			3.8	6.4	89.7
EUCAST-TVL n)	**1**		**1**	**3**	**2**	**9**	12.5	31.3	56.3
- participants n)	**7**			**34**		**92**	5.3	25.6	69.2

To the authors’ knowledge, there are no publicly available statistics on the AST standards utilized in laboratories. At least for RV-A, we determined the EUCAST standard to be overwhelmingly applied nowadays, while only a few participants still utilize the CLSI standard being the most applied standard back in 2012 (Figure 4B).

### 3.5 Adherence to AST recommendations of a German guideline on uncomplicated urinary tract infections

**FIGURE 5:**
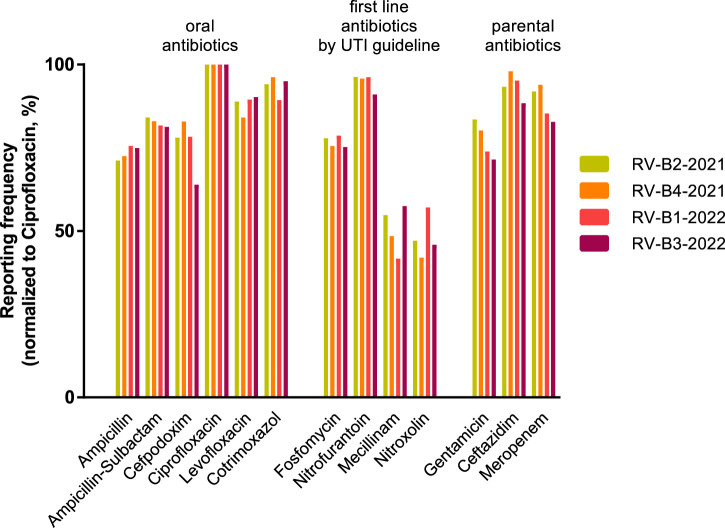
Reporting of urology guideline-recommended antibiotics in RV-B. Reporting frequencies of specific antibiotic substances for *Escherichia coli* normalized for ciprofloxacin rates as 100% in four individual RV-B rounds in 2021 and 2022. Substances are grouped into oral and parental antibiotics as well as antibiotics recommended by a German S3 guideline for uncomplicated urinary tract infections as first-line therapy.

Finally, we investigated how the efforts of medical guidelines were supported by the AST of participants in RV-B. A German urology S3 guideline on uncomplicated urinary tract infections updated in 2017 recommends the antibiotics fosfomycin (single oral dose), nitrofurantoin, nitroxoline, and pivmecillinam for premenopausal women to counteract the constant development of resistance, particularly to fluoroquinolones in the field of urology (Kranz et al., 2018). According to EUCAST, these substances are only to be evaluated in full for *E. coli*. The reported AST for this species in RV-B is therefore a measure of the guideline adherence of the participating laboratories. Reported results for 4 *E. coli* strains sent out in RV-B in 2021 and 2022 were analyzed. We set the most frequently tested antibiotic per round–ciprofloxacin - equal to 100% and found nitrofurantoin (mean value 94.8%), fosfomycin (76.8%), mecillinam (50.6%), and nitroxolin (48.0%) less frequently reported compared to other oral or parenteral antibiotics (Figure 5). Hence, roughly half of RV-B participants did not report on two first-line antibiotics, while the medical society representing most of them recommends their therapeutic use.

### 3.6 Accession of EQAS management comments

The comments written by the EQAS management for each test round are available for download on the Instand e.V. website after the EQA certificates have been issued. We checked the accession rate of these comments that had been available for at least 3 months at the end of 2023. An average of 19.8% (range 2.9%–56.9%) of participants overall accessed the comments, while the difference between an average of 35.7% of RV-A participants, but only 13.7% of RV-B participants was striking. On the other hand, the comments are probably also of interest to laboratories that did not participate in the respective EQAS: 13.4% of downloads for RV-A and 27.8% for RV-B were made by users who did not participate in the respective EQAS round.

## 4 Discussion

Analyzing the data obtained by managing the two bacteriology EQAS RV-A and RV-B we recognized the overall high standards in bacteriological diagnostics in Germany with passing rates for specialized laboratories in recent years of 95% or higher (Figure 1B). Previous data from the respective Swiss EQAS from 1992 until 1996 showed comparable results (Siegrist et al., 1998), while analysis of EQA in other countries, especially in developing ones, tended to show lower passing rates. Moreover, three of these studies show educational effects in terms of improvements over time (Chaitram et al., 2003; Perovic et al., 2019; Wattal et al., 2019), while our data, and a study on bacteriology in the Eastern Mediterranean Region, with higher failure rates, hardly detected improvements (Squires et al., 2022). Even if the overall diagnostic accuracy is on a high level, EQAS always have the chance to send out certain bacteria unveiling limitations and directions of improvement; especially by including species, that have come into medical focus only recently and for which correct identification or AST might be not well established in laboratories.

The public health system greatly benefits from this highly reliable diagnostic level, as it serves, as a data basis for epidemiological developments, is crucial in detecting and containing local outbreaks, and guides towards an effective but specific antibiotic therapy. However, the comparability of EQAS data and studies involving human clinical microbiology data, in general, is low and in need of standardized reporting (Turner et al., 2019).

Technological progress is one strengthening aspect in this regard. The identification of bacteria using Matrix Assisted Laser Desorption Ionisation - Time of Flight (MALDI-TOF) has become of great importance for microbiological diagnostics during the study period (Figure 2D). It is considerably faster than biochemical reactions and largely independent of the correct selection of a system suitable for a defined germ spectrum. In addition, the reliability of identification, especially in routine operations, is raised to a previously unknown level. However, the use of the MALDI-TOF method requires a relevant investment that can only be made by larger laboratories. As a consequence, there has been a steady increase in its use in the specialized laboratories participating in RV-A, while its use in the predominantly smaller, specialty-specific, and outpatient-providing laboratories participating in RV-B has remained at a comparatively low level. Only the use of the highly automated Vitek system, which is widely available in the majority of laboratories due to the sensitivity tests carried out with this system, still accounted for a higher proportion of identifications for a relatively long time. Irrespective of the method, our data shows that identification of frequently found bacteria species is more successful compared to rarely found ones recapitulating findings of EQAS in other countries (Wonglumsom et al., 2008; Wattal et al., 2019) (Tables 2, 3). In terms of consistent communication of microbiological reports, the use of current terminology is a desirable goal (Figure 3). A laboratory’s constant effort to keep up with this development, which is considerably more dynamic due to molecular biological analyses, is essential for this. In addition, the implementation of the current nomenclature depends to a large extent on the implementation of changes in commercial identification systems by the respective manufacturers.

While the identification of bacterial species remained on a very low failure rate during the observed period, two major modifications in the requirements for successful AST increased the failure rates significantly (Figure 4A). Evidence-based medicine is generating more and more reliable and species-specific data sets on bacterial infections and minimal inhibitory concentration (MIC)-distributions (Leclercq et al., 2013; Kahlmeter, 2015). Correlating these *in vivo* findings with the *in vitro* AST, led to and will further result in an increased amount of breakpoint tables and recommendations in the different standards issued by expert committees. Laboratories have to navigate this development and adhere to a certain standard to evaluate obtained MICs on a good data basis. In this regard, major hurdles leading to failing in the AST category have been recognized. i) Incorrect results for an individual substance caused by technical errors, reagents of insufficient quality, or errors in reading are found to be major aspects not only in this study but also in others (Perovic et al., 2019). ii) Utilizing an outdated version of the reported AST standard leads to failures in evaluations (e.g., categorical result “I", although not defined according to the standard or evaluation of substances no longer seen as applicable for the bacterial species) (Wattal et al., 2019). In this regard, suppliers from commercially available AST systems need to implement updates promptly to enable consistent interpretation not only in terms of AST standards but also concerning updated treatment guidelines (Figure 5). iii) Moreover, substances, for which no specific breakpoints are listed but evaluation can be derived from indicator substances, are not reported (e.g., cefoxitin screen for staphylococci for oxacillin, cefuroxime, ampicillin-sulbactam) and or an incorrect selection of antibiotics is chosen. In particular, since the introduction of antibiotic selection by participants, up to one-third of participants failed the susceptibility testing part due to an insufficient number of antibiotics tested. These findings are in line with the observation of Perovic et al. analyzing the African EQAS concerning AST (Perovic et al., 2019).

In Germany, microbiological laboratories are legally obliged to participate in EQAS. However, they are not obliged to adhere to a specific AST standard. Therefore, the EQAS is the only instance to monitor and evaluate AST utilization (Figure 4B). Nearly all laboratories participating in RV-A applied to the EUCAST standard, which is favorable for public health as this increases the comparability of results and AST evaluations. However, the CLSI standard is still used by a minority of EQAS participants, which can lead to different reports regarding AST. This trend in standards utilization is also found in other European countries (Altorf-van der Kuil et al., 2017; EUCAST international uptake, 2024).

The example of an NDM-expressing *P. mirabilis* indicates the associated variability besides an already existing technical variation of measurements (Table 7). This circumstance makes it difficult to compare the results obtained, at least for individual antibiotics, and thus to communicate them for therapeutic or epidemiological purposes and to projects recording antibiotic resistance developments, like Antibiotic Resistance Surveillance (ARS) managed by Robert Koch Institute (RKI, Berlin, Germany) for instance (ARS - Antibiotika-Resistenz-Surveillance, 2007; Walter et al., 2017). As a consequence, existing AST standards concerning the secure and sensitive detection of epidemiologically relevant resistance mechanisms should be followed thoroughly.

AST standards define how to detect specific resistance mechanisms, however, consistent reporting standards on these are not defined. Hence, reporting of resistance mechanisms can only be assessed in the EQAS on a voluntary basis. For public health applicable standards and subsequent evaluation of the adherence to them in EQA seems as a future goal in light of increasing antimicrobial resistance rates worldwide. Methicillin-resistant staphylococci, vancomycin- or linezolid-resistant enterococci, and ESBL- or carbapenemase-producing Enterobacterales must be identified with the highest degree of certainty to prevent further spread in the healthcare system. While a high degree of reliability is achieved in the determination of the phenotype (evaluation as “R") for prominent resistances in staphylococci (methicillin) and enterococci (vancomycin), the labeling of pathogens as MRSA or VRE is only rarely carried out in RV-B and is likely reflecting daily laboratory reports in an outpatient setting (Table 5).

The aforementioned aspects all indicate the importance of an EQAS focusing on bacteriology for reliable individual diagnostics but also subsequent public health on a bigger scale. Nonetheless, we recognized a decline in the number of participants in RV-B starting around 2014, while RV-A showed more constant participation (Figure 1A). This could be related to the increased quality assurance requirements for laboratories stipulated in the legally binding directive given by the RiliBÄK (Bundesaerztekammer, 2023), but also to an improved offer from specialized laboratories to private practitioners and finally to changes in remuneration leading to a consolidation in the bacterial diagnostics market. The studied EQAS cannot claim to be exhaustive as it is not the only bacteriological EQAS on the market, but the represented number of laboratories both in RV-A and RV-B is representative of the German field of bacteriology.

The issued comments on each EQAS round give the chance to emphasize developments or methodological pitfalls and have thereby the chance to support the spreading of knowledge and indirectly support public health. This fact is recapitulated by the accession not only by participating laboratories in the respective EQAs round but also by registered non-participants.

## Data Availability

The data analyzed in this study is subject to the following licenses/restrictions: Individual EQAS results are confidential. Requests to access these datasets should be directed to SZ, stefan.ziesing@mh-hannover.de.
